# Community perceptions on the effectiveness of herbal medicines and factors associated with their use in managing diarrhoea among under-five children in North-eastern Tanzania

**DOI:** 10.1186/s41182-023-00537-5

**Published:** 2023-08-25

**Authors:** Edwin Liheluka, Nyasiro Sophia Gibore, John P. A. Lusingu, Samwel Gesase, Daniel T. R. Minja, Maike Lamshöft, Denise Dekker, Theodora Bali

**Affiliations:** 1 Department of Public Health and Community Nursing, University of Dodoma, Dodoma, Tanzania; 2 Department of Educational Psychology and Curriculum Studies, University of Dodoma, Dodoma, Tanzania; 3 National Institute for Medical Research, NIMR, Tanga Centre, Tanga, Tanzania; 4 Bernhard Nocht Institute for Tropical Medicine, BNITM, Hamburg, Germany; 5 German Center for Infection Research, DZIF, Hamburg, Germany; 6 University Hospital Hamburg-Eppendorf, Hamburg, Germany

**Keywords:** Herbal medicines, Diarrhoeal diseases, Under-five children, Paediatric health workers, Caretakers of under-five children, And traditional healers

## Abstract

**Background:**

The demand for herbal medicines continues to increase globally. However, community perceptions on their effectiveness and factors influencing their use have not been extensively investigated, notably in the Tanga Region, North-eastern Tanzania, where their use in treating various diseases, including paediatric diarrhoea, has flourished. According to studies, Tanga Region has a high prevalence of diarrhoea among under-five children. This study explored community perceptions on the effectiveness of herbal medicines and factors associated with their use in managing diarrhoea among under-five children in North-eastern Tanzania.

**Methods:**

A qualitative approach and a narrative design were employed by the present study since they had the potential to reveal unrecognized or unreported research problems. Focus group discussions and in-depth interviews were used to facilitate data collection from June 2022 to February 2023. The methods were chosen since they are the most common sources of qualitative data in health research. Purposive sampling method was used to select 247 participants, which included 171 caretakers, 52 traditional healers, and 24 paediatric health workers. Interviews were conducted until the saturation point was reached. The purposive technique was considered since it was a method that enabled the researcher to select participants who were knowledgeable about the study topic. Data analysis was performed using thematic analysis.

**Results:**

Economic hardship, culture and heritage, superstitious beliefs, failure to recover after receiving hospital medication, easy accessibility of herbal medicines, and long distance to the health facility were the factors perceived to be potentially associated with persistent use of herbal medicines among caretakers. The majority of participants believed that herbal treatments were harmless and effective in treating diarrhoea.

**Conclusion:**

Superstitious beliefs, culture, and heritage were the primary justifications for using herbal medicines. It is vital for the relevant authority to educate the community on the risk of using unproven herbal medicines in order to diminish the effects that may arise from using uninvestigated herbs. As things stand, the use of herbal medications will continue owing to their relevance to the lives of people in the study setting.

## Introduction

Specific plant-based harvests have been utilized for managing and treating various ailments for centuries in almost every setting across the world [1]. Even with the progressive endorsement of conventional medicines, the use of herbal medicines in treating various ailments has grown supremely all the way through [2]. For example, about 60–90% of the population in non-industrialized countries utilizes herbal medicines for prevention and treatment of both communicable and non-communicable ailments [2, 3]. In the same way, the mounting utilization of herbal medicines for preventive and palliative care has been proclaimed even in industrialized countries [2, 3]. In Brazil, Japan and China for example, various herbal medicines have been researched and approved for human use [2].

In most non-industrialized settings, herbal medicines have retained their esteem owing to the historical convergence that existed for a long haul [4–6]. The rationale cited for using herbal medicines, particularly across underdeveloped nations, includes belief and attitude towards the herbs, detachment from the health facility, and a severe shortage of health workers, drugs, and medical equipment. Additionally, safety, efficacy, aspiration for self-care, easy accessibility of herbal medicines, and barrier-freeness of herbal medicines have been reported to influence their use [1, 7].

Diarrhoea is the most important public health burden at a global scale. According to statistics, there are over 1.7 billion cases of paediatric diarrhoeal illnesses each year. [8]. In 2016, for instance, diarrhoeal ailments were graded as the 8th leading cause of death, accounting for more than 1.6 million fatalities [9]. Evidence from previous studies shows that approximately 30% of diarrhoeal-related deaths occurred among children aged less than five years, and nearly 90% of deaths occurred in sub-Saharan Africa and South Asia [9, 10].

In Tanzania, diarrhoeal ailments among children aged less than five years are prevalent predominantly during the rainy season and are associated with age, education, and the substandard socioeconomic status of caretakers [11]. While literature has indicated that nearly 60–79% of Tanzanians make use of herbal medicines in treating various ailments, including diarrhoea, diarrheal illnesses are still prevalent in the country [2].

Studies conducted in Tanzania and other settings across the world revealed that herbal medicines have been perceived to have the required therapeutic effects capable of completely curing diarrhoeal illnesses for both adults and children [12–15]. It is believed that there are still communities that rely solely on herbal medicines to treat various diseases, including diarrhoeal diseases, with full recovery without the need to even visit a health facility [16]. This outlook somehow bears out that herbal medicines can possibly heal various ailments, although more scientific evidence is still required [17, 18]. On the other hand, the long-standing use of herbal medicines has aroused worries about public health due to the periodic inclusion of unethical behaviours’ that disregard patients' safety [19]. It is important to note that, despite the fact that many herbal medicines are thought to offer therapeutic advantages in treating a variety of disorders, the majority of these herbs have never been thoroughly examined and authorized for use in humans, and their usage is often regarded as random [20].

It has been suggested that since ancient times, herbal medicines have played an incredibly decisive role in battling various and complex ailments that have been troubling the majority of people globally [4, 19, 21]. Having figured out the contribution and reputation of herbal medicines in serving nearly 80% of the world's population, the World Health Organization (WHO) is presently developing a traditional medicine strategy (2014–2023) that aims to yield scientific guidelines that will pilot the meaningful delivery of herbal medicine services across the world [2]. In Tanzania, traditional and alternative medicine practices are governed by Act No. 23 of 2002 [22]. The endorsement of this law was the government's opening move in rolling out alternative health care services in Tanzania [22]. The act was basically set up in order to oversee and regulate the provision of services delivered by traditional healers (THs), traditional birth attendants, and traditional medicine sellers [22].

The use of herbal medicines is very common among under-five children with diarrhoea, especially in rural settings [12–14]. It is alleged that the increasing utilization of herbal medicines relevant for treating childhood diarrhoea is an integral component of the long-standing culture that communities have inherited from previous generations [1, 19, 21]. In the Tanga Region the use of herbal medicines to cure a variety of illnesses is quite popular [23, 24], while at the same time, diarrhoea prevalence among under-five children is also high. Figures show that in 2019 and 2020, the prevalence was 60% and 59%, respectively [25].

Prior studies conducted in the Tanga Region were unable to clearly identify the additional factors that led to the region's significant usage of herbal treatments [24]. There is a claim that the Tanga Region is home to an abundance of medicinal plants; possibly this explains why people choose to utilize herbal medicines to cure a variety of illnesses, although this claim has not been adequately explored by prior studies. This study was thus carried out to find out what additional factors influence caretakers in the region, particularly in the districts of Korogwe and Handeni, to favour utilizing herbal medicines to cure their under-five children. This study also attempted to understand how the public felt about the efficacy of herbal medicines in treating diarrhoea among under-five children. Gathering data in this area was essential for the sake of making a more accurate assessment of how herbal medical practises might work in conjunction with other modes of formal healthcare delivery to combat diarrhoeal illnesses in the local community and beyond.

The present study has been able to identify the rationale behind why so many individuals strongly embrace herbal medicines for curing various ailments, including diarrhoeal infections among under-five children. It was found that communities’ positive perceptions, beliefs about herbal medications and the long-standing cultural linkages between herbal medicines and the people in the study setting have led to widespread use of herbal therapies. The findings of this study need to serve as an eye-opener for the relevant health authorities to monitor trends in the use of herbal medicines, the majority of which have never been well studied to determine their therapeutic efficacy in treating and eradicating pathogens of various illnesses, including diarrhoea, in under-five children. Furthermore, the findings of this study should encourage a thorough botanical investigation to determine whether or not the herbal medicines reported in the present study have anti-diarrhoeal potentials. If it is determined that these treatments have anti-diarrhoeal effects, the appropriate authorities should think about advising that they undergo clinical trials to determine their safety and efficacy before being eventually licensed for use in humans.

## Research methodology

### Study setting

The present study was conducted in Korogwe and Handeni districts, Tanga Region, North-eastern Tanzania from June 2022 to February 2023. The Korogwe and Handeni districts have been earmarked for this research since the majority of the population considerably embodies the practices of utilizing herbal medicines for management of varied ailments, including diarrhoea [23, 24]. Korogwe District has a population of 359,421, while Handeni District has a population of 493,321 [26]. The inhabitants of Korogwe and Handeni districts are mainly Sambaa and Zigua. There are other tribes as well (Bondei, Pare, Maasai, and Nguu), but these form the minority group. The main economic activities in the two districts include petty trading in the urban areas and subsistence farming as well as livestock keeping in the rural areas [27, 28].

### Study design and approach

A narrative research design and a qualitative study approach were employed to explore two specific study objectives, which were:

1. To explore community perceptions on the effectiveness of herbal medicines in managing diarrhoeal ailments among under-five children in Korogwe and Handeni districts, North-eastern Tanzania.
2. To explore community perceptions on factors associated with the use of herbal medicines in managing diarrhoeal ailments among under-five children in Korogwe and Handeni districts, North-eastern Tanzania.

A qualitative approach and a narrative study design were chosen since they enabled the researcher to gain a more comprehensive and in-depth understanding of the study topics from information-rich participants. In addition, through purposive sampling, the researcher could choose participants (key informants) who had adequate knowledge and experience about the study topic [29, 30]. To our knowledge, a study of this nature has never been done in the districts of Korogwe and Handeni, so it was crucial to purposively select key informants who have made it possible to achieve the research goals.

In addition, the combination of IDIs and FGDs for data gathering allowed this study to triangulate its techniques of collecting data. Furthermore, this research has included participants of different backgrounds, experiences, and knowledge regarding diarrhoeal diseases and herbal medicines who were caretakers of under-five children, paediatric health workers, and THs, something that was vital in establishing the trustworthiness of the study results. For instance, the approach and design have made it easier for the researcher to comprehend the justifications that motivate caretakers to use herbal medicines to treat diarrhoeal illnesses among their under-five children.

### Study population

The study population included caretakers of under-five children, paediatric health workers, and THs in Korogwe and Handeni districts. The caretakers were chosen since they were using herbal medicines to treat their under-five children, while paediatric health workers and THs were responsible for managing diarrhoeal cases by using modern and herbal medicines, respectively.

### Inclusion criteria

- Caretakers of under-five children attended 12 health facilities earmarked for this study in Korogwe (seven) and Handeni (five) districts, and they were registered in the District Health Information System (DHIS) book.
- Traditional healers with more than one year of experience in treating under-five children in Korogwe and Handeni districts.
- Paediatric health workers (clinicians and nurses) who were working in 12 health facilities earmarked for this study in Korogwe and Handeni districts.
- Male and female adults (18 years old and above) who consented to participate in the study.

### Exclusion criteria

- Participants who were ill and unable to participate in the study.
- Absentees (participants) on the day of data collection.

### Data collection methods

Focus group discussions (FGDs) and In-depth Interviews (IDIs) were employed in this study to facilitate data collection. The methods were preferred for the reason that they are the most common source of qualitative data in health research [31]. A combination of FGD and IDI methods of data collection (triangulation) was employed to ensure the reliability of the data.

### Sampling technique of study participants and health facilities

Purposive sampling technique was used to select FGD and IDI participants and 12 health facilities. The choice of technique was considered for the reason that it is a technique that is broadly preferred in qualitative studies as it allows the researcher to select and include participants in a specific study who are knowledgeable and experienced about the study topic [31]. In the present study, participants were selected based on their roles, as explained below.

### Selection of IDI participants

The present study recruited 24 paediatric health workers, 24 caretakers whose under-five children had experienced diarrhoea within the past six months before the interview date of this study, and 52 THs, as explained hereunder.

Korogwe and Handeni districts have a total of 10 divisions, with four divisions in Korogwe District and six in Handeni District [32, 33]. Within the two districts, there are 12 main health facilities (four hospitals and eight health centres). The present study recruited participants from each of the 10 divisions and each of the 12 main health facilities in the districts. The aim was to gather a wide range of viewpoints from stakeholders across both districts. Twelve main health facilities serve most of under-five diarrhoeal patients in the two districts; that’s why they were selected for this study.

### Selection of paediatric health workers

Two health care workers (one paediatric clinician and one paediatric nurse) were purposively selected from each of the 12 main health facilities to participate in this study. Paediatric health workers were selected since they were treating children with diarrhoea. Furthermore, they were aware of the different types of treatment that under-five children receive.

### Selection of caretakers

Two caretakers (a female and a male caretaker) were purposively selected from each of the main 12 health facilities to participate in this study. Registered caretakers in the DHIS book who brought their under-five children to the health facility for diarrhoeal treatment within the past six months before the interview date of the present study were selected. The research team identified participants from the DHIS book at the health facility and traced them in the community before they were sensitized and invited for the interview. The DHIS book includes a variety of patient information, such as name, place of residence, gender, age, and the disease that brought the patient to the health facility for treatment. Caretakers were included in this study for the reason that they were the ones who decide what kind of treatment to seek for the child suffering from diarrhoea.

### Selection of THs

The present study purposively recruited 52 THs from each of the 10 divisions found in Korogwe and Handeni districts. The TH coordinators of Korogwe and Handeni districts were consulted to select a minimum of four and a maximum of six THs from each division to participate in the present study. Traditional healer coordinators managed to select five THs from each of the eight divisions in Korogwe and Handeni districts. Two divisions, Mombo in Korogwe District and Mkumburu in Handeni District, provided six THs since they were the divisions with the largest number of THs, which made the number, reach 52. The criteria for selecting THs were their experience and reputation in treating children within Korogwe and Handeni districts.

### Selection of FGDs participants

Eligible caretakers for this study were those who brought their under-five children to the health facility for diarrhoea treatment within the past six months prior to the interview date for the present study and were registered in the DHIS book. The research team identified participants from the DHIS book at the health facility and traced them in the community before they were sensitized and invited for the interview.

### Familiarization and sensitization meetings before the interviews

After participants were identified and selected, the Principal Investigator (PI) (EL), and research assistants located the participants physically (face by face) where they live and explained to them that they have been selected to participate in the study. The research team also took that opportunity to enlighten each participant about the study objectives and the importance of their participation in the study. Similarly, the PI (EL), obtained contact details for each participant such as mobile phone number, village and sub-village name and arranged the interview date with them, which significantly facilitated communication before, during and after the interviews. These meetings by far, enabled the research team to build rapport with the participants which greatly assisted the implementation of this research. All the participants who were invited to the interview attended possibly, because of the well informative sensitization meetings that were done before the study started, except for only nine participants who were invited to the FGDs but did not attend. Three participants had an emergency of illness and six participants travelled outside the study area.

### Conducting IDIs

The PI (EL), with assistance from research assistants, conducted all the IDI interviews. The interview process started once a respondent agreed to participate in the study and signed and dated the Informed Consent Form (ICF). Data were recorded by one research assistant by taking notes, and after the participant agreed, the digital audio-visual recorder was used. The research assistant was responsible for recording the interviews using the digital audio recorder under the supervision of the PI (EL). One IDI took approximately 20–35 min to complete. Only one researcher the PI (EL) interviewed an IDI participant at a time, while a research assistant took notes. The IDIs were carried out in a place convenient to the participant. Transport costs to the meeting venue were reimbursed for all study participants.

### Conducting FGDs

The present study conducted a total of 24 FGDs, and each group comprised a minimum of six and a maximum of ten participants. Twelve groups were for male caretakers, and 12 groups were for female caretakers. The PI (EL), with support from research assistants, conducted all the FGD interviews. The interview process started after FGD participants agreed to participate in the study and signed and dated the ICF. Data were recorded by research assistants by taking notes, and after getting approval from participants, a digital audio-visual recorder was also used. A research assistant was accountable for recording the interviews by using the digital audio recorder under the supervision of the PI. One FGD took 25–45 min to complete. The PI (EL) interviewed FGD participants while a research assistant took notes. The venue for FGDs meetings was organized with assistance from the local village leaders. Transport costs to the meeting venue were reimbursed for all study participants.

### Data management and analysis

Data analysis was performed using thematic analysis. The method was selected for its flexibility, which enabled the investigator to produce the most recent concepts that have been derived from the collected information [34]. The data were collected in Kiswahili and later transcribed verbatim and translated into English. Finally, the analysis was carried out following the six phases of thematic analysis suggested by Braun and Clarke: familiarization with the data, generating first-round codes, searching prospective themes, reviewing themes, defining and naming themes, and ultimately generating the report [34].

Familiarization with the data: The data gathered were adapted to understand the richness of the information in relation to the study objectives. In this step, the PI and an experienced qualitative researcher repeatedly perused and scrutinized the data for the sake of grasping the scope of the arguments discussed during the interview with potential study participants. After grasping the wealth of the collected data, the PI (EL), with the help of two research assistants and an experienced researcher differently started to identify potential codes that were finally used during final report writing.

Generating first-round codes: In this step, beginning codes useful to study objectives were sketched and systematized into a sensible category. The focus was to establish core elements that aided in connecting what was reported by the participants in relation to the research objectives. During this process, the PI (EL), with the help of two research assistants and an experienced researcher differently started off to establish core categories that helped to connect what was reported by participants in connection to the study objectives.

Searching prospective themes: After the data have been properly skimmed through the whole data package, the PI (EL), with the help of two research assistants and an experienced researcher, independently structured dissimilar codes into prospective study themes and put together suitable covert data across the categorized study themes.

Reviewing themes: At this phase, the themes were examined separately by the PI (EL), with the help of two research assistants and an experienced qualitative researcher to determine if they had satisfactory supporting data. After critically reviewing the themes, other themes were tailored and combined.

Defining and naming themes: At this phase, after the PI (EL), identified potential themes that had sufficient supporting data with the help of two research assistants and an experienced qualitative researcher, the themes were scanned, assessed, and polished further for the sake of making them ready for final data analysis. Finally, before generating the report, the study PI and an experienced qualitative researcher went through the analysis process, which was done separately by the two researchers step by step, and agreed that the themes rendered were based on the data collected from the participants. Six themes emerged during the analysis, which included economic hardship, culture and heritage, superstitious beliefs, failure to recover after being treated by hospital medicines, easy accessibility to herbal medicines, long distances to the health facility, and the perceived efficacy of herbal medicines in treating diarrhoea. Finally, the report-writing process began by following the sequence of the arranged themes.

### Trustworthiness of study process and data

The trustworthiness of the present study is in accordance with the credibility, transferability, dependability, and confirmability of the study results, which have been obtained after the study team physically, met and conducted face-to-face interviews with all the participants who took part in this study.

Credibility: “refers to the assurance in the genuineness of the study results obtained” [35]. By being able to execute the proposed scientific research methods, single out appropriate respondents, giving respondents the freedom to participate or not to participate in the study after explaining the aim of the study, and carrying out a member check model, the findings of this study are credible as recommended by the credibility decree [36].

Transferability: “refers to the circumstances of showing that study results obtained from one setting can also be obtained in a different setting if conducted by a different researcher” [35]. In order to ensure the results that have been obtained from this study can also be obtained elsewhere if conducted by a different researcher, the present study categorically described the sample size and data collection methods that were employed. Additionally, the present study clearly stated the inclusion and exclusion criteria of the respondents who participated in this study, as well as the study setting and the organization (The University of Dodoma) that oversaw the conduct of the study. The present study adhered to all the above criteria, which are key in determining the validity of the study findings should other research of this kind be conducted elsewhere by a different researcher [36].

Dependability: “refers to the circumstances of demonstrating that the study results are dependable and could be repeated if conducted in similar circumstances” [35]. For this to be achieved, the present study implemented what was initially proposed during study proposal development. In addition, the study managed to sufficiently describe the entire flow of the research conduct from the beginning to the very end until the results were obtained. By being able to wholly describe the study’s conduct and implement what was proposed in the study protocol, the findings of this study are dependable, as recommended by the dependability decree [36].

Confirmability: “refers to the extent of objectivity to which study conclusion is produced by what participants have reported and not the investigator’s curiosity” [35]. In order to ensure confirmability, the present study demonstrated how data were collected after sensitizing the study communities, and the verbatim quotes used in the study resulted from FGD and IDI interviews. The quotes were extracted from the exact transcripts, which were personal narratives from study participants [36].

## Results

A total of 247 individuals participated in this study, which included 171 caretakers, 52 THs, and 24 paediatric health workers. A total of 147 participants (caretakers) took part in the FGDs, and 24 participants also caretakers took part in IDIs. Additionally, 52 THs and 24 paediatric health workers participated in IDIs. Caretakers and THs ages ranged from 18 to 67 years (Table 1). The majority of caretakers and THs participants had primary education. Furthermore, study results show that male participants across all groups attained relatively higher levels of education compared to females. The main occupation for most caretaker participants was subsistence farming and petty trading. Nearly all THs reported doing healing work as their primary occupation. Clinicians and nurses were the paediatric health workers who participated in this study. Their age ranged from 25 to 67 years; 17 (71%) were female and seven (29%) were male, all of whom had attained college or university education (Table 1).

**Table 1:** Socio-demographic characteristics of study participants

Socio-demographic characteristics of participants (*N* = 247)			
Characteristics	Caretakers *N* = 171 (69%)	Health care providers *N* = 24 (10%)	Traditional healers *N* = 52 (21%)
District			
Korogwe	102	14	21
Handeni	69	10	31
Sex			
Male	88	7	32
Female	83	17	20
Age (in years)			
18–40	114	16	10
41 >	57	8	42
Data collection methods			
In-depth interviews	24	24	52
Focus group discussions	147	0	0
Level of education			
No formal education	11	0	7
Primary education	116	0	41
Secondary education	37	0	4
College/university	7	24	0

The findings of this study are presented under the following key themes, which emerged during the analysis of the interview transcripts: *economic hardship, culture and heritage, superstitious beliefs, failure to recover after being treated by hospital medicines, easy accessibility of herbal medicines, long distance to the health facility, and efficacy of herbal medicines in treating diarrhoea.*

### Economic hardships

The majority of participants across all groups indicated that financial hardship forced the majority of caretakers to make use of herbal medicines. Participants, particularly health workers, revealed that notwithstanding the claim that treatment for children under the age of five is free of charge, there are medicines and other services that need to be paid for. But also, participants, especially caretakers, claimed that sometimes you find that the health facility is pretty far away and you don’t have transport fare to get to the hospital, and as a matter of fact, you can get to the hospital and still find that even those medicines are not available. Therefore, people ponder it is more rational to benefit from the readily available herbal medicines:

*“If you go to the hospital, you will spend four thousand shillings for transport fare* (~1.71USD)*, and yet you will be told that medications have run out of stock, so you will have to buy them, and at the same time, you don’t have money. That is why our elders told us to use certain herbal plants, and others will go and pick them for you and instruct you how to use them, and by God’s mercy, the child recovers”.* FGD No 10, P No 56, Caretaker, Female

*“The first reason is that you cannot afford hospital expenses. The second reason is that you know if I give the child this herbal medicine, it will help him”.* FGD No 01, P No 01, Caretaker, Male

In accord with caretakers, THs had this to narrate:

*“The first reason is the difficult economic situation; you find a parent does not have money to pay for hospital treatment, so he uses alternative medicine first in order to solve the child’s diarrhoeal problem”.* IDI, P No 1, Traditional Healer, Male

*“Other people decide to use herbal medicines because they have no transport fare. Thus, they attempt to use herbal medicines while finding money for hospital treatment, but surprisingly enough, God might help them to recover with herbal medicines”.* IDI, P No 24, Traditional Healer, Female

Health workers also agreed with what were said by caretakers and THs by stating:

*“Sometimes the reason is the cost of hospital treatment as not all medicines are freely given for children, so he tells you I was told to buy medicine and I had no money, that’s why I decided to use herbal medicine, but they didn’t help him, so it seems some caretakers do not afford hospital treatment costs”.* IDI, P No 21, Clinician, Male

*“For the time that I have worked in the Tanga Region and looking at the challenges of the people here, for most people the issue is income; for some, it's their geographical location where they are located and the challenges of getting to the health facilities. Let’s say a large percentage of the population is in a poverty situation, so they are looking at what will be the first aid for the child. So, they use herbal medicines, and some trees are widely known in the Tanga area; there are herbs called Mzumbasha (Ocimum suave), and some use Guava leaves (Psidium guajava)”.* IDI, P No 13, Clinician, Female

### Culture and heritage

It was learned from the participants that the use of herbal medicines has a historical connection with the traditions and customs of the people that they have inherited over the years, when there were no alternative medicines to nurse the sick. According to the study participants, it sounds as if such linkage will hold out for generations to come as long as humans continue to live. Moreover, the participants, predominantly caretakers and THs, have asserted that herbal medicines are deeply rooted in their diurnal lifestyles and that their use is a noteworthy aspect of their lives. Participants had these to delineate:

*“Another reason is that our ancestors were treated with herbal medicines; for example, since my grandfather was born, he has started using hospital medications just recently; even if it is a wound, he usually picks up the leaves of a medicinal plant and puts them on the wound, and it dries up”.* FGD No 2, P No 08, Caretaker, Female

*“I use these herbal medicines because even my grandparents were using them, and I have been using them since my childhood. We have grown up in a herbalist family, so when our grandparents used herbs to treat diseases, we observed and became familiar with herbal practises.” *FGD No 3, P No 12, Caretaker, Male

In agreement with caretakers THs had this to reveal:

*“It’s the culture of our community to use herbal medicines because these medicines have existed since the days of our ancestors. That’s why if a person goes to the right and gets stuck, he has to turn to the left”.* IDI, P No 16, Traditional Healer, Female

*“The use of these medicines is in their traditions and customs”.* IDI, P No 16, Traditional Healer, Female

In support, the health workers reiterated:

*“Other times it is just beliefs, because in the past people used those medications and they were healed. So, they decide to use them as a quick action; they start using herbal medicines at home, but when they discover that the medication is not working, that's when they will rush to hospitals.” *IDI, P No 14, Nurse, Female

*“It is because of their belief in herbal medicines”.* IDI, P No 22, Nurse, Female

### Superstitious beliefs

Overall, diarrhoea as a condition appeared to be well known among study participants, including caretakers and THs. By a considerable margin, the most commonly cited cause of diarrhoea among children aged less than five years, mainly by caretakers and THs, was superstitious beliefs. Interestingly, all caretakers and THs who cited superstitious belief as a cause of diarrhoea believed hospital medicines are completely incapable of treating this type of diarrhoea, and therefore, herbal medicines are the only treatment option capable of treating it. For example, caretakers had this to explain:

*“The hospital medicines do not treat zongo* (a famous name meaning someone has been bewitched)*. You go to a herbalist, and he gives you medicine that your child licks and recovers. Even mtula plant (Solanum incanum); you pick seven leaves of mtula (Solanum incanum)—you cover one leaf, you open the next leaf until reaching seven leaves, you mix with water, then you boil them and give them to the child".* FGD No 14, P No 83, Caretaker, Female

*“If you have been bewitched with zongo, you have to take a certain medicine where you will purge (through diarrhoea or vomiting). So, we use herbal medicines to solve problems like zongo”.* FGD No 03, P No 9, Caretaker, Male

Traditional healers endorsed what was debated by caretakers by intimating:

*“There is a type of diarrhoea that needs a natural remedy; there is the so-called zongo, and there are other ailments that cause a person to have diarrhoea because there is an evil person who caused it by magic, so we provide medicine for that”.* IDI, P No 4, Traditional Healer, Male.

*“They use herbal medicines because they know that there are various diseases that hospital medications cannot cure but herbal medicines do; for example, zongo”.* IDI, P No 3, Traditional Healer, Female

The sentiments of health workers did not contradict those given by caretakers and THs; certainly, the community strongly believes that there is a type of diarrhoea caused by witchcraft (popularly known as bewitched with Zongo); however, they as health professionals do not believe in that:

*“Many caretakers think their children have diarrhoea because they have been bewitched with zongo. They say the child will die if he is bewitched with zongo and then uses hospital medicines, and indeed, they say if you inject a child, he will die. Therefore, the antidote of zongo is herbal medicine, so it is a belief”.* IDI, P No 2, Nurse, Female

*“They are being treated with herbal medicines because I always ask them what they have given their children before coming to the health facility, and many say I took him to a traditional healer. You know in this community there is what they call zongo, and if they see a child with diarrhoea, they believe it is due to zongo”.* IDI, P No 21, Clinician, Male

### Failure to recover after being treated by hospital medicines

The majority of participants, primarily caretakers and THs, were of the view that failure to recover after using hospital medicines is the justification that motivates caretakers to make use of herbal medicines. Traditional healers predominantly decreed that herbal medicines are superior in treating diarrhoeal diseases and many other diseases, presumably more than modern medicines. On the part of the health workers, they thought that the fear of being hospitalized is the rationale that motivates caretakers to use herbal medicines. Caretakers had this to describe:

*“Yes, even my child had severe diarrhoea, and I took him to the hospital, where he was injected with drips of water, but he did not recover. Therefore, I was told by my aunt to take the leaves of the cashew nut tree, I ground and filtered them, and gave them to the child; I did so, and diarrhoea stopped”.* FGD No 02, P No 12, Caretaker, Female

*“When my child was at the age of teething, he had diarrhoea for a very long time; I took him to the hospital, but he did not recover. Therefore, I took him to a TH. He gave me the medicines that he had already made, so I did not know from which tree they were extracted. My child used the medicines and recovered”.* FGD No 07, P No 40, Caretaker, Female

In addition, THs elaborated:

*“You find that the child has had diarrhoea for a long time, and the caretaker bought pills and gave them to the child without getting better, and he knows that if I take a certain herb and give it to the child, he will recover.”* IDI, P No 40, Traditional Healer, Female

*“They decide to use herbal medicines after using hospital medicines and they did not recover or they took a long time to recover, so they use herbal medicines to solve the problem of diarrhoea quickly”.* IDI, P No 09, Traditional Healer, Male

In contrast, health workers were of these views:

*“Others think that if I go to the hospital, the child will be hospitalized”.* IDI, P No 15, Clinician, Female

*“Another reason is fear of being hospitalized”.* IDI, P No 15, Nurse, Female

### Easy accessibility of herbal medicines

Participants suggested that herbal medicines are easily accessible since they are just wild plants that are not cultivated by anyone. They can simply be located on their farms, in the forest, by the roadsides, or even just outside the house. Therefore, due to their easy accessibility, the majority of people have seen them as an alternative to modern medicines that are given in hospitals where caretakers have complained so much that they are expensive:

*“A large percentage of these herbal medicines are found right here in our environment”.* FGD No 13, P No 77, Caretaker, Male

*“They are found within our environment”*. FGD No 12, P No 70, Caretaker, Female

In agreement with caretakers, THs were of these views

*“Most of them are found in our environment.”* IDI, P No 44, Traditional Healer, Female

*“They are found close by. But especially when it’s an urgent patient case, you must prepare them first, because you may not be able to get the leaves close by because the drought has already arrived. So, when we have a surplus of leaves, we prepare so that we can store the medication”.* IDI, P No 37, Traditional Healer, Male

When the health workers were asked about the accessibility of herbal medicines, they had this contribution:

*“They decide to use herbal medicines because they are easily found in the environment, they live in; they get advice from their elders, either grandmother or aunt”.* IDI, P No 01, Clinician, Male

*“Guava plants are found in our surroundings because they are fruit trees”.* IDI, P No 18, Nurse, Female

### Long distance to the health facility

All groups of participants mentioned this theme. The participants were of the view that appreciable distance to the health facility, especially in isolated communities, is one of the key motivations that contribute to the use of herbal medicines for the majority of caretakers of children aged below five years. During the discussions, the participants were able to clearly show that the unavailability of health facilities near people’s settlements creates an enabling environment for the community to bring to light alternative health care services, especially in times of emergencies.

For example, caretakers had this to say:

*“Our hospital is far away; so, we use herbal medicines to help the sick child for a while, and we look on how his condition will be. If he gets better, we continue to use it, and if he doesn’t get better, then we take him to the hospital”.* FGD No 3, P No 14, Caretaker, Male

Another participant echoed:

*“The reason is the distance from home to the hospital, as my colleague said. A child may start having diarrhoea at nine or four o’clock in the night. So, you look for alternative treatment and observe what his condition will be in the morning”.* FGD No 3, P No 13, Caretaker, Male

Traditional healers as well concurred with the arguments made by caretakers:

*“Others decide to use herbal medicines because the hospital is far away”* IDI, P No 24, Traditional Healer—Female

*“Distance also contributes to the decision to use herbal medicines, and sometimes the illness occurs at night and the parent cannot go to the hospital during that time.”* IDI, P No 46, Traditional Healer—Male

The health workers also agreed indisputably with what was articulated by the caretakers and THs:

*“The first reason, I think, is distance. Most people who use herbal medicines are those who live very far away, because most of the people we treat here at Kideleko come from very far away. There are Maasai, if they will tell where they are from; to get there, it's very far, so they are not able to afford the cost of bringing their children here whenever they are sick. They will only come to the hospital when the sickness is severe”.* IDI, P No 24, Nurse, Female

*“Others decide to use herbal medicine because the hospital is far away”* IDI, P No 15, Clinician, Female

### Efficacy of herbal medicines in treating diarrhoeal diseases

The effectiveness of herbal medicines became an important theme that all participants had something to talk about. The majority of caretakers and THs specifically praised the wonders of herbal medicines in successfully treating diarrhoeal-related illnesses, as they supposed that herbal medicines have the required therapeutic properties to completely cure diarrhoeal diseases, and the caretakers would no longer need to go to the hospital. However, the majority of health workers were very skeptical about the efficacy of herbal medicines; they bolded that the herbs have not adequately been researched and their use is illogical since they are medicines without specific doses; even the mixing formula is not clearly understood either.

For example, caretakers had this to say:

*“Herbal medicine is very good because it treats quickly and effectively, and even our ancestors were using it”.* FGD No 1, P No 02, Caretaker, Male

*“I started with hospital medications, and they failed, but when I used herbal medicines, he got better”.* FGD No 08, P No 43, Caretaker, Female

A few caretakers were of different opinions. They suggested that herbal medicines are like first aid, and caretakers must visit the health facility after taking herbal medicines regardless of the child’s recovery or not, as explained below:

*“We use it as first aid when the child has diarrhoea, but we must take him to the hospital”.* FGD No 14, P No 80, Caretaker, Female

*“I prefer hospital treatment most because it is very difficult to know what problem the child is suffering from, and when he cries, you cannot rush to pick herbal medicines and give them to him; you must take him to the hospital so that doctors can examine his problem and give him appropriate medication”*. FGD No 17, P No 100, Caretaker, Male

Traditional healers were of these views:

*“These medicines have the ability to treat very well, sometimes even better than hospital medicines”.* IDI, P No 08, Traditional Healer, Male

*“For herbal medicines, even when a child is severely ill, when you give them, they will get relief very quickly”.* IDI, P No 40, Traditional Healer, Female

In contrast, health workers had this to express:

*“Because I don’t know the contents of these medicines and I don’t know if research has been conducted to confirm if they are effective or not, I cannot be sure if they have the potential to treat diarrhoea. You know some diseases are self-limiting, so the caretaker may believe that the child has taken herbal medicines and recovered while diarrhoea has stopped on its own because it is a self-limiting disease*.” IDI, P No 21, Clinician, Male

*“According to my understanding, herbal medicines cannot treat a child who has diarrhoea. Maybe if they are researched, they can help us in the future, but right now what I know is that they cannot help. However, if I say we should discourage the use of herbal medicines, it would be wrong, but I advise those involved with herbal medicines to bring those medicines so that they can be researched, and if they are proven to be helpful, we will use them. But if people continue to use them without proper doses, that is dangerous for human health”.* IDI, P No 22, Nurse, Female

Some health workers thought that, to a certain extent, herbal medicines are curative; that's why people keep using them, and besides that, some hospital medicines have been derived from plants:

*“They can treat because even our modern medicines come from herbal medicines, so there are herbs that heal. For example, we use folic acid as a blood-boosting supplement, but there are leaves that stimulate blood production twice”.* IDI, P No 17, Clinician, Male

*“There are children who use herbal medicines and recover completely because you find they have been treated on time, but diarrhoea will persist if it is caused by other reasons, but if it is due to a temporary stomach upset, herbal medicines can treat it”.* IDI, P No 08, Nurse, Female

Regarding adverse effects, nearly all participants, especially caretakers and THs, believed that herbal medicines for diarrhoeal diseases have no side effects. A few participants, particularly health professionals, certain caretakers, and THs, were, however, of the opinion that, if used improperly, herbal medications might have adverse effects:

For example, caretakers explained that:

*“I have never experienced any side effects”. FGD No 1, Participant No 04, Caretaker, Male*

*“Herbal medicines have no side effects”. FGD No 18, Participant No 108, Caretaker, Female*

In agreement, the majority of THs thought herbal medicines for healing diarrhoeal illnesses in children under five are completely safe and appropriate for human usage. The participants reported that they had never received any negative feedback about herbal medicines' side effects since they began using them to treat diarrheal diseases. Participants could express the following, for instance:

*"Ever since I started doing this work, I haven’t seen any side effects. I don’t know it in a more professional way. But I have never received a patient who got too much dosage, was unconscious, or overdosed. This has never happened”.* IDI, Participant no 44, Female, Traditional healer

*“They do not have side effects. A wild tree cannot be harmful”.* IDI, Participant no 21, Male, Traditional healer

Contrary to the majority of THs and caretakers, all health professionals and a small number of caretakers and THs considered that herbal medicines have some adverse effects to some level since they are treatments that have not been scientifically established to be effective for human usage:

*“They don't have established dosages, and their expiration date is also unknown, so they could potentially have side effects,”* IDI, Participant No 03, Nurse, Female

*“They don't have specific doses, so I think they have side effects.”* IDI, Participant No 21, Clinician, Male

In agreement, caretakers had this to narrate:

*“If you don't utilise them as directed, I believe there may be adverse effects.” FGD No 01, Participant No 02, Caretaker, Male*

Despite their effectiveness, some herbal medications, according to a few THs, might have minimal adverse effects if the patient doesn't follow the directions for using the medication:

*“If you don't take the recommended dosage, these medicines may have adverse effects; this is true even with hospital drugs”.* IDI, Participant No 01, Male, Traditional healer

## Discussion

This study explored community perceptions on the effectiveness of herbal medicines and factors associated with their use in managing diarrhoea among under-five children in North-eastern Tanzania. The findings are discussed below with reference to other studies that have been carried out in Tanzania and other diarrhoeal-endemic countries.

Exaggerated concerns with respect to the expenditure associated with diarrhoeal treatment, such as transport and drug expenses in health facilities, were noted among the participants. Particularly, the caretakers firmly suggested that their limited financial resources motivate them to use herbal medicines to cure their under-five children who were suffering from diarrhoea and/or other illnesses. Similar findings were also reported in studies conducted in Nigeria and South Africa [4, 5]. In support of the caretakers’ theme, the majority of health workers and THs also believed that economic hardship among the majority of caretakers in the study area largely encouraged and forced them to utilize herbal medicines. This caretaker’s perspective on hospital expenses may be due to a limited knowledge of the current recommendations for paediatric medical care, since in Tanzania, treatment for children under the age of five should ideally be provided without charge [37]. It is important for the relevant authorities to take appropriate actions, reach out to the caretakers through different platforms, such as the media, and make them understand that health services for under-five children are free. Caretakers therefore do not need to be frightened to use these services, as it is their fundamental right, per Tanzania’s health policy. It has been documented that public health education through radio, television, social media and the like is fundamental in enlightening the community about various health-related matters [38, 39].

Distance to the health facility was also reported by the majority of participants across all groups as one of the rationales that motivate caretakers to make use of herbal medicines, which is similar to a number of other studies from around the world [4, 40]. This claim could be accurate given that the study setting is marked by scattered mountain ranges, apexes, and river basins, and that the main economic activities for the bulk of the inhabitants there are farming and raising livestock [27, 28]. In light of this authenticity, the majority of people live in isolated areas where they can comfortably find enough land for their economic deeds. However, residing in distant places would inevitably lead many individuals to be disconnected from areas with access to medical amenities. Although the Tanzanian government aspires to build a dispensary in every village, at the moment government health facilities are largely only present at the ward level and in a few villages [37]. Given that the communities in the study settings tend to migrate sometimes in pursuit of livestock pastures and locations for agricultural operations; it is actually complex for the government to build a health facility in every village. As a result, community members are urged to reside in recognized villages with access to medical facilities.

Easy accessibility of herbal medicines to places where people live has been voiced as one of the reasons that motivate caretakers to make use of those medicines. This subject matter may be rational owing to the fact that Tanga Region is blessed with a great wealth of biological biodiversity, which is an important source of medicinal plants [23, 24]. The appropriate authorities may think about carrying out botanical studies in order to gather proof about the reliability, excellence, and effectiveness of these herbal medicines.

Responses across all groups of participants implied that herbal medicines are a crucial element that societies have retained throughout olden times. Studies conducted in Nigeria, Saudi Arabia, and Jordan also reported similar findings [3, 4, 7]. Due to the fact that the use of herbal medicines is ingrained in the culture of the people in the study setting, the use of these unscientifically backed medications is likely to continue for a long time to come. Therefore, in order to mitigate the use of unproven anti-diarrhoea herbal medicines, the relevant authority has to take another look at Tanzania’s current health policy and consider how it might take into consideration the cultures of the people in different settings. As it is, it appears that the present health policy has not given much thought to the matter of people's cultures, which may explain why the use of unproven anti-diarrhoea herbal medicines has flourished. Studies have shown that it is critical to take into account local customs when developing various health policies or planning a particular intervention since any effort that ignores or undermines local culture may not be at all successful [41–43].

The study’s results also demonstrate that the participants, particularly caretakers and THs, had a considerable belief that there are instances where a child might get diarrhoea for reasons that are not medical but rather owing to a superstition-based basis. Studies conducted in Nepal and South Africa also reported similar findings [40, 44, 45]. This attitude among caretakers and THs could be due to a lack of facts on the biomedical causes of diarrhoeal diseases, which in turn affects their health-seeking behavior at the health facility level. Such an attitude may contribute to a delay in seeking formal health care for prompt correction of dehydration and electrolyte imbalances that may lead to death in the community. Contrary to this finding, the WHO documented that diarrhoea is caused by an assortment of bacteria, viruses, and parasites resulting from eating contaminated food or drinking contaminated water [8]. Continuous civic health education, especially in rural areas can aid the community to better understand several themes linked to health [38, 39].

Regarding the effectiveness of herbal medicines, study results indicate that caretakers and THs in particular were of the opinion that patients' failure to improve after receiving hospital treatment is another rationale for caretakers' use of herbal medicines. Some THs and caretakers were adamant that herbal medicines were superior to contemporary medications for treating diarrhoea and other illnesses. Studies carried out in Ghana, Saudi Arabia, and Nigeria revealed similar results [3, 4, 13]. In contrast, the majority of healthcare professionals were not convinced that herbal medicines were more effective than conventional ones in treating diarrhoea. They suggested that perhaps the fear of being hospitalized is what drives caretaker to use herbal medications. A tiny group of medical professionals, however, nevertheless held the belief that herbal medicines could treat diarrhoea, arguing that if they were unable to do so, they would not have been widely utilized. This health worker’s attitude may be a result of the information their caretakers imparted to them when they were young about the use of herbal medicines in curing a variety of diseases. There is a need for more rigorous research to be conducted in order to validate whether or not herbal medicines reported by participants in Handeni and Korogwe districts have ant-diarrhoeal properties.

With regard to adverse effects of herbal medicines, the majority of THs and caretakers believed that herbal medications used to treat diarrhoeal diseases among under-five children were completely safe for humans. In contrast to the beliefs of all other health professionals who believed that herbal medications can have some negative adverse effects, although they lacked any evidence to support this claim. For example, THs maintained that since they began prescribing herbal medicines to under-five children, they have never witnessed any child being harmed by them, and, as a result, they assume the medicines have absolutely no negative consequences. It's interesting to note that while almost all THs were extremely knowledgeable about herbal medicines for diarrhoeal illnesses, they were less knowledgeable about the medicines’ possible side effects. This unawareness learned from caretakers and THs might have been parented by the lesser degree of analysing diverse elements broadly given that most of them had basic formal education and a few had never even attended formal school [46].

Contrary to these results, research carried out elsewhere revealed that herbal treatments for diarrhoeal diseases can have negative consequences such as tiredness, nausea, lassitude, vomiting, stomach discomfort, black tongue, tinnitus, abortion, and liver issues in living organism [15, 47]. It is important for the relevant authority to keep a watchful eye on anyone who fails to recognize the dangers of using untested herbal medicines. The authorities should think about developing long-term plans to regularly inform the public about the risks associated with using untested herbal treatments. As anticipated, the majority of health professionals, a few caretakers, and a very small number of THs considered that herbal medicines may have adverse effects because the bulk of the medicines had not been scientifically proven to be safe for use in humans. This state of awareness for some participants is promising since it demonstrates that society is starting to grasp important health matters.

### Lesson learnt

Despite loads of rationales that have been declared as motivating factors for caretakers to make use of herbal medicines, the study findings have established that the use of herbal medicines will continue because of its connection with the lives of people since when mankind began to live. This matter of truth can be validated from the assumption that there are segments of people with economic prospects in urban areas where there are all advanced health facilities but still the same group of people strongly believe in herbal medicines [21, 48].

In addition, study result revealed that, the safety of most herbal medicines is still unknown, even though they are widely used to treat unhealthy people. Nearly all participants, particularly caretakers and THs, believed that herbal medicines were safe; they also witnessed that they had never experienced any side effects since their childhood when they started utilizing them. This testimony alone from the participants should not be enough to justify the slapdash use of these medicines. Therefore, there is a need to carry out more systematic studies in order to substantiate their safety and efficacy.

## Conclusion

The study demonstrated that culture and heritage, easy accessibility of herbal medicines, patients’ failure to recover after being treated with modern medicines, economic hardship, superstitious viewpoints, and the distance to the health facility were the factors reported to be potentially associated with persistent use of herbal medicines among caretakers in North-eastern Tanzania. On the other hand, it was found that herbal medicines were perceived to be efficacious enough to treat diarrhoeal ailments. However, the quality, safety, and efficacy of all herbal medicines reported in the present study are still unknown. Therefore, it is rational for the relevant authority to reflect on conducting controlled research for the sake of providing more scientific evidence related to the usefulness and risks of herbal medicines that are used in managing diarrhoeal ailments. Well-researched and regulated herbal medicinal products can possibly boost the income of herbalists while simultaneously ensuring that patients receive the best and efficacious herbs and that the government can also collect revenue.

## Data Availability

The dataset generated and/or analyzed in this study is available from the corresponding author on reasonable request.

## Abbreviations

DHIS: District Health Information System
FGDs: Focus group discussions
ICF: Informed Consent Form
IDIs: In-depth interviews
PI: Principal Investigator
THs: Traditional healer/s
UNICEF: United Nations Children’s Fund
UDOM: University of Dodoma
IRRC: Institutional Research Review Committee
WHO: World Health Organization
